# Development of a Dietary Factor Assessment Tool for Evaluating Associations between Visceral Fat Accumulation and Major Nutrients in Japanese Adults

**DOI:** 10.1155/2019/9497861

**Published:** 2019-02-13

**Authors:** Hideto Takase, Naoki Sakane, Toshihisa Morimoto, Takanobu Uchida, Kenta Mori, Mitsuhiro Katashima, Yoshihisa Katsuragi

**Affiliations:** ^1^Biological Science Research, Kao Corporation, 2-1-3, Bunka, Sumida-ku, Tokyo 131-8501, Japan; ^2^Division of Preventive Medicine, Clinical Research Institute, National Hospital Organization Kyoto Medical Center, 1-1, Fukakusamukaihata-cho, Fushimi-ku, Kyoto 612-8555, Japan; ^3^Consumer Products–Human Health Care Business, Kao Corporation, 1-14-10, Nihonbashi Kayaba-cho, Chuo-ku, Tokyo 103-8210, Japan; ^4^Health Care Food Research, Kao Corporation, 2-1-3, Bunka, Sumida-ku, Tokyo 131-8501, Japan

## Abstract

**Background and Objectives:**

The increased prevalence of metabolic syndrome necessitates the establishment of tools for evaluating dietary factors associated with visceral fat accumulation and preventing visceral fat obesity. Here, we aimed to develop a dietary factor assessment tool for evaluating visceral fat accumulation.

**Methods:**

We conducted a dietary habit questionnaire survey and visceral fat measurement by bioelectrical impedance analysis in 11,438 adults (Survey 1) and a dietary habit questionnaire survey and dietary assessment based on 3-day meal records in 579 adults (Survey 2). Dietary habit factors were identified by factor analysis with varimax rotation, and their relationship with visceral fat accumulation and major nutrients were analyzed.

**Results:**

Factor analysis of the dietary habit questionnaire revealed the following five main dietary factors: “Appetite (15 questions),” “Healthy food choice (5 questions),” “Sedentary behavior (6 questions),” “Calorie restriction (5 questions),” and “Irregular mealtime (4 questions).” “Appetite” correlated positively with visceral fat accumulation and energy intake mainly from carbohydrate. “Healthy food choice” correlated negatively with visceral fat accumulation and positively with the protein/fat ratio, dietary fiber/carbohydrate ratio, and N-3 fatty acid/fat ratio. Dietary guidance to modify excess energy intake and increase nutritional balance might be effective toward preventing visceral fat accumulation.

**Conclusions:**

The dietary factor assessment tool developed in this study can be used to diagnose problems related to dietary habits and provide guidance for dietary modifications aimed at preventing visceral fat accumulation.

## 1. Introduction

Metabolic syndrome is defined as the clustering of obesity with a number of associated metabolic disorders. Because patients with metabolic syndrome are at risk for developing serious health disorders, such as diabetes and atherosclerotic disease, prevention and improvement of metabolic syndrome have become global issues [1–3]. Each country has different criteria for diagnosing metabolic syndrome as there are multiple sets of standards; in Japan, visceral fat accumulation is considered an essential condition [4] given its association with disease incidence irrespective of simple weight loss or gain. In this context, treatment for obesity or improvement of metabolic syndrome should focus on the reduction of visceral fat and prevention of visceral fat accumulation.

For the treatment and improvement of obesity, lifestyle modifications, particularly dietary habits, are crucial, and various dietary guidelines and methods aimed at improving obesity and nutritional balance have been proposed [5–8]. Dietary methods targeting visceral fat reduction, however, are not well established due to difficulties in measuring visceral fat by X-ray, computed tomography (CT), or magnetic resonance imaging when conducting large-scale studies. A device using bioelectrical impedance measurement to evaluate visceral fat accumulation was recently developed [9], making it easier to study dietary habits and visceral fat accumulation in a large population.

Dietary habit modification for obesity improvement requires an assessment tool to evaluate dietary habits and nutritional balance at an individual level. Several questionnaires to assess the relationship between dietary habits and obesity have been developed [10–12], including the Eating Behavior Scale (EBS) for Japanese adults [13], but these questionnaires are primarily used for evaluating dietary habits associated with obesity. Within this context, the development of a dietary habit questionnaire that allows for assessment of dietary habits focusing especially on visceral fat accumulation was required. Recently, Yanagisawa et al. developed a dietary habit questionnaire comprising 35 representative questions that was statistically validated [14]. Accordingly, we conducted a survey combining this questionnaire and visceral fat measurements using a bioelectrical impedance visceral fat meter to identify dietary factors associated with visceral fat accumulation (Survey 1). In addition, a survey combining the questionnaire and 3-day dietary records was also conducted to clarify the dietary factors and their nutritional values (Survey 2).

## 2. Methods

### 2.1. Survey 1: Dietary Habit Survey and Visceral Fat Measurement

#### 2.1.1. Participants and Recruitment Methods

This survey targeted male and female employees of 15 companies and 42 offices in Japan that cooperated in this survey who were at least 20 years of age and enrolled in their company's health insurance union. The number of survey participants was targeted to more than 10,000 to guarantee a sufficient number to reflect the Japanese working age population. Participants were recruited via posters and workplace communications (e.g., intranet) announcing visceral fat measurement meetings at each workplace. Employees who wished to participate in the survey received written and verbal explanations of the contents of the survey, and those who provided informed consent on a voluntary basis were included in the survey population. This study was reviewed and approved by the ethics review board of National Hospital Organization Kyoto Medical Center and conducted in accordance with the ethical considerations outlined in the Declaration of Helsinki. Survey 1 was conducted between 2011 and 2013.

#### 2.1.2. Survey Items

Visceral fat measurements were performed using a bioelectrical impedance visceral fat meter (EW-FA90; Panasonic Corporation, medical device approval No.: 22500BZX00522000) at the umbilical level during gentle exhalation in the standing position. If there was marked fat accumulation and the umbilicus was displaced downward, measurements were performed at the midpoint between the lower border of the ribs and the anterior superior iliac spine. Measurements were performed by medical staff who received sufficient training on the measurement method, and the average value of three or more measurements was calculated. The use of this measuring instrument allows for simultaneous measurement of waist circumference and visceral fat area. Height and weight were also measured, and body mass index (BMI) was calculated.

The dietary habit survey was conducted using the newly reported dietary habit questionnaire comprising 35 items assessed on a 5-point scale. Participants were asked to select one of the following five choices: 1, not applicable; 2, not very applicable; 3, neither; 4, somewhat applicable; and 5, applicable.

### 2.2. Survey 2: Dietary Habit Survey and Dietary Assessment

#### 2.2.1. Participants and Recruitment Methods

This survey was conducted by a contract agency (Macromill, Inc.) targeting men and women 30 to 65 years of age residing in the Tokyo metropolitan area and registered with the survey company. Because visceral fat accumulation showed an increase in the 30s or later in the interim analysis of Survey 1, the age of the participants in Survey 2 was planned to be in the 30s or older and the number of participants in each age group was aimed to be equal. The sex ratio of the participants was 70% male and 30% female to reflect the interim analysis of Survey 1. To ensure the accuracy of the nutritional assessment, the total number of participants was targeted to 600, so that each sex and age group would contain more than 40 participants. Participants were recruited by e-mail announcements sent to monitoring members. Individuals interested in participating in the survey received a written explanation of the survey contents via a personal online survey page, and those who provided informed consent on a voluntary basis were included in the survey population. Ethical considerations regarding the survey were given according to the code of ethics of the survey company and abided by the rules of personal information protection. Survey 2 was conducted between 2011 and 2012.

#### 2.2.2. Survey Items

**Figure 1:**
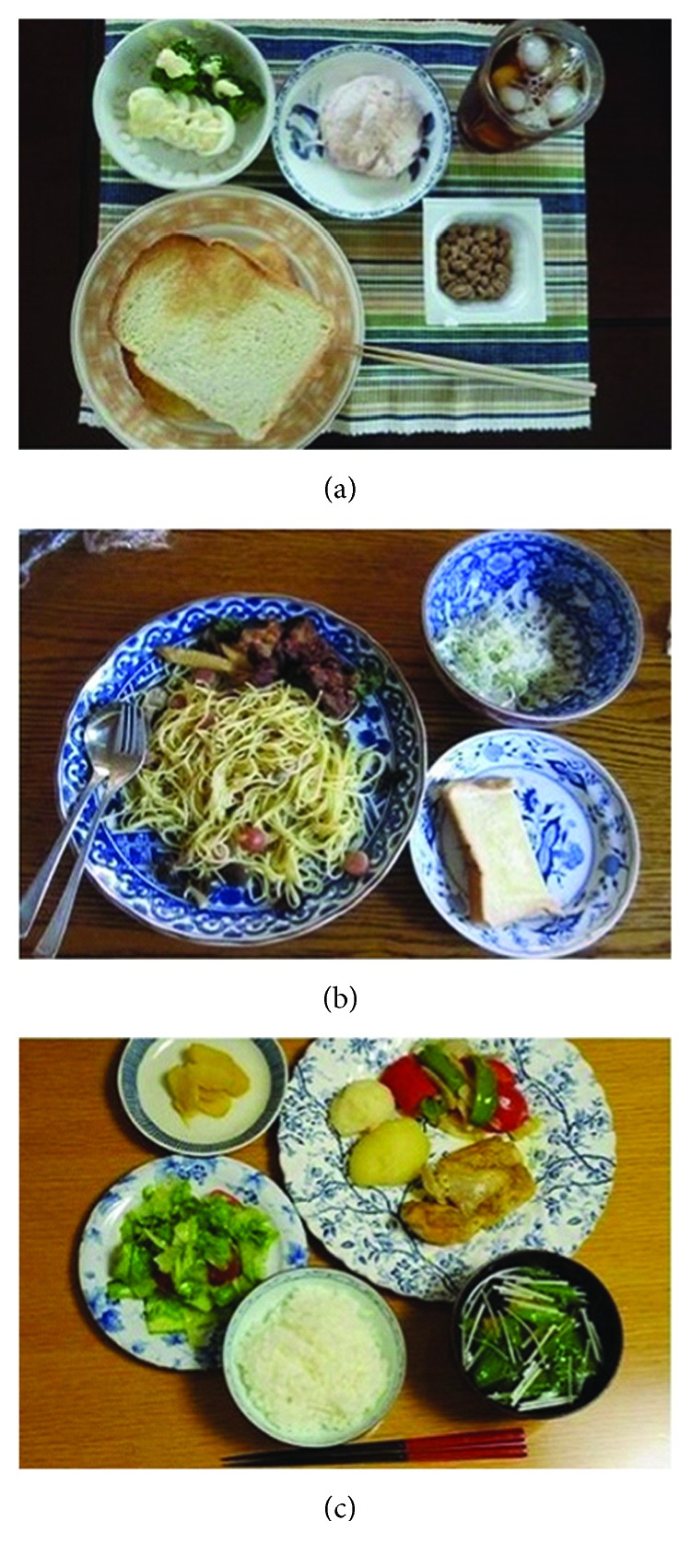
Samples of uploaded photographs.

The dietary habit survey was conducted similarly to Survey 1 by displaying the dietary habit questionnaire on an Internet website. In addition, participants were asked to photograph all meals, including snacks and beverages, for three consecutive days starting on a nonworking day and upload them from their personal online survey page. When photographing meals, as a rule, they were asked to capture standard size dishes (including chopsticks and spoons, etc.) within the same photograph to facilitate estimation of the size of food items. Samples of uploaded photographs are shown in Figure 1. For all meals, the contents, approximate amount, meal start/finish time, and place (e.g., home, restaurant, workplace, etc.) were also recorded. When participants missed a meal, this was recorded accordingly.

Uploaded photographs of meals were subjected to food group and nutritional composition analysis by a nutritionist trained in dietary analysis according to the Standard Tables of Food Composition in Japan, Fifth Revised and Enlarged Edition [15]. The Standard Table of Food Composition in Japan has been revised to the seventh edition in 2015, but we adopted the fifth edition, the latest version of the time when the Survey 2 was conducted. The analysis was performed using dietary composition analysis software (Healthy Maker Pro 501; Mushroom Soft, Co., Ltd.) that contains a database of typically consumed Japanese meals and foods. Age, height, weight, and abdominal circumference of each participant were obtained from self-reported values entered in the survey page, and physical activity intensity levels were investigated with the International Physical Activity Questionnaire (Short version) [16, 17].

### 2.3. Statistical Analysis

**Figure 2:**
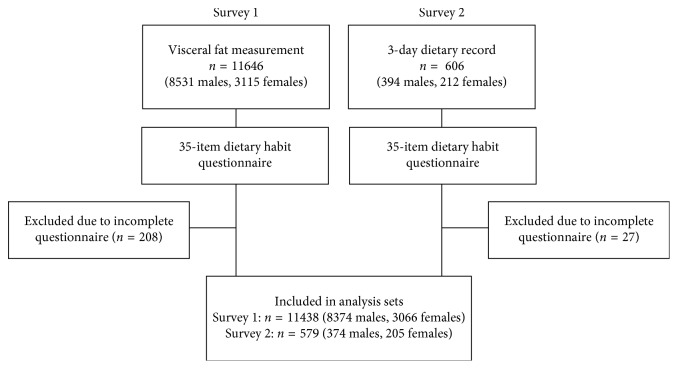
Flow chart of survey participants.

In Survey 1, interim analyses were conducted sequentially during the survey period (2011–2013), and eventually visceral fat measurements were performed in 11,646 participants (8531 men and 3115 women). Of these, 11,438 (8372 men and 3066 women) who completed the dietary habit questionnaire were included in the analysis. In Survey 2, the 3-day meal survey was conducted with 606 participants (394 men and 212 women), and 579 (374 men and 205 women) of these who completed the dietary habit questionnaire were included in the analysis (Figure 2).

Physical characteristics of participants in Surveys 1 and 2 are shown as mean ± standard deviation. The analysis of the dietary habit questionnaire was performed by combining responses from both surveys. Questionnaire responses were analyzed by factor analysis with varimax rotation. Among the obtained factors, scores of five factors that were interpretable as dietary habits with an eigenvalue ≥1 and potentially associated with obesity were calculated. The scores of these five factors were divided into quartiles. In Survey 1, BMI and visceral fat area adjusted by sex and age were calculated and compared by quartiles of each dietary habit factor score. Similarly, in Survey 2, characteristics of dietary conditions related to each dietary habit factor were compared by quartiles of each dietary habit factor score. Comparison among quartiles was performed by analysis of variance (ANOVA). Prior to the ANOVA, equality of variance among quartiles was checked by Levene's test. If the equality of variance among quartiles was not observed, data were log-transformed.

All statistical analyses were performed using IBM SPSS Statistics 24 (Armonk, NY, USA), with a significance level set at 0.05 (two-tailed test).

## 3. Results

### 3.1. Characteristics of Survey Participants

Table 1 shows the physical attributes of participants in Survey 1 and Survey 2 by sex and age. The male/female balance in Surveys 1 and 2 was 73%/27% and 65%/35%, respectively. Although there was a significant difference in BMI in the male participants in their 30s, there was no significant difference between Survey 1 and Survey 2 in BMI by other sex or age groups.

### 3.2. Dietary Habit Analysis

Table 2 shows eigenvalues and factor loadings of dietary habit factors calculated by the factor analysis of the dietary habit questionnaire. The identified factors were “Appetite,” “Healthy food choice,” “Sedentary behavior,” “Calorie restriction,” and “Irregular mealtime” based on the content of questions with a large factor loading. Comparison of the dietary factor score between survey, sex, and age groups are shown in Table 3. As expected, there were significant differences in dietary habits between the sex and age groups. This indicates that adjustment of sex and age was necessary to analyze the relationship between eating habits and obesity in this research. Dietary factor score was not significantly different between Surveys 1 and 2, except for “Appetite.” Therefore, eating habits related to appetite must be compared cautiously between the surveys.

### 3.3. Survey 1: Dietary Habit Survey and Visceral Fat Measurement

Table 4 shows sex- and age-adjusted BMI and visceral fat area according to quartiles of each dietary factor score. There was a significant difference in visceral fat area across quartiles of each factor score (ANOVA, *p* < 0.01 for all), with Q4 showing a significantly larger visceral fat area compared with Q1, except for scores of “Healthy food choice.” As for “Healthy food choice,” Q4 (i.e., the highest factor score) showed a significantly smaller visceral fat area. On the other hand, while a significant difference was observed in BMI across the quartiles of each factor score, the difference in BMI between Q1 and Q4 was not significant for “Healthy food choice.”

### 3.4. Survey 2: Dietary Habit Survey and Dietary Assessment

We analyzed the associations between dietary habit factors and actual dietary conditions related to these factors by quartiles of dietary factor scores in Survey 2 participants.  Table 5 shows dietary intake by food group and major nutrients according to quartiles of “Appetite” factor score. By food group, significant differences were observed across quartiles in the amount of intake of “mushrooms” and “beverage,” (ANOVA, *p* < 0.05). Intake amounts for “sugar and sweetener” and “egg” was significantly higher in Q4 compared to Q1. Consequently, significant differences were observed in calorie intake and carbohydrate intake across quartiles, with significantly higher calorie intake, fat intake, and carbohydrate intake in Q4 compared with Q1.

Table 6 shows the characteristics of dietary intake according to quartiles of the “Healthy food choice” factor score. By food group, significant differences were observed across quartiles in the amount of intake of “beans,” “vegetables,” “seaweed,” “fish and seafood,” and “seasoning and spices” (ANOVA *p* < 0.05). Intake amounts of “beans,” “vegetables,” “fish and seafood,” and “seasoning and spices” were significantly higher in Q4 compared with Q1. Furthermore, no difference was observed in calorie intake across quartiles, while significant differences were observed in protein, N-3 fatty acid, EPA + DHA, dietary fiber, and salt intake (ANOVA *p* < 0.05). Compared with Q1, Q4 showed significantly higher intakes of N-3 fatty acid, EPA + DHA, dietary fiber, and salt.

Table 7 shows the characteristics of physical activity intensity according to quartiles of “Sedentary behavior” factor score. The Q1 group, which had a low “Sedentary behavior” factor score, showed significantly higher total physical activity intensity, but this was attributed to a longer time of moderate to vigorous activities. No difference was observed in walking time.

Table 7 shows intake amounts of main nutrients according to quartiles of the “Calorie restriction” factor score. Calorie intake was significantly lower in Q4 compared with Q1, but this was attributed to the lower carbohydrate intake, and no differences were observed in protein and fat intake. Table 7 also summarizes mealtime data, which relate to the quartiles of “Irregular mealtime” factor score (i.e., factor related to mealtime). While there was no difference in the start time of breakfast or lunch with respect to “Irregular mealtime,” the group with a higher “Irregular mealtime” factor score had a significantly delayed start time of dinner, with a high rate of skipping breakfast.

## 4. Discussion

In this study, we aimed to elucidate dietary habits associated with visceral fat accumulation and to clarify actual dietary conditions using a newly reported dietary habit questionnaire. Dietary habits were classified and quantified as five factors (i.e., Appetite, Healthy food choice, Sedentary behavior, Calorie restriction, and Irregular mealtime) using the questionnaire. The scores of these factors and visceral fat area were compared across quartiles, and dietary habits associated with visceral fat accumulation were analyzed. Factor scores for “Appetite,” “Sedentary behavior,” “Calorie restriction,” and “Irregular mealtime” contributed positively to visceral fat accumulation, whereas the factor score for “Healthy food choice” contributed negatively. As these factor scores can be obtained individually according to each respondent's dietary habits, the questionnaire will allow for assessment of dietary habits to determine which factors contribute to visceral fat accumulation at an individual level. In this context, it is a promising tool for making suggestions for dietary habit modification on an individual basis.

By interpreting each dietary habit factor score in combination with visceral fat accumulation and actual dietary conditions, more detailed suggestions could be provided for dietary modification. The group with a high “Appetite” factor score consumed more “sugar and sweetener” and “beverage,” i.e., food items that are not part of a meal. This group had a high calorie intake mainly due to increased carbohydrate intake. These results suggest that for those with visceral fat accumulation and a high “Appetite” factor score, dietary guidance that focuses on limiting excess carbohydrate intake, such as “sugar and sweetener” and “beverage” might be effective. Although there was no significant difference, snacks intake increased roughly 2.5 times in Q4 compared to Q1 and limiting snack intake might also be effective.

The group with a high “Healthy food choice” factor score tended to consume more “beans,” “vegetables,” “fish and seafood,” etc., despite the constant intake of calories and had significantly higher intakes of protein, dietary fiber, and N-3 fatty acid. These findings suggest that nutritional balance could be improved and the accumulation of visceral fat prevented by modifying food selection without reducing calories consumed. Differences in BMI across quartiles of the “Healthy food choice” factor score were not significant (Table 4). As the “Healthy food choice” factor does not affect the increase or decrease of calorie intake, its impact on weight gain/loss, which depends on energy balance, might be small. In reality, however, if we provide dietary guidance to simply increase the intake of protein and N-3 fatty acid, calorie intake itself would increase. Moreover, because many food items that are rich in dietary fiber are also rich in carbohydrates, calories consumed would inevitably increase. Therefore, based on the ratios of various nutrients under conditions of constant calorie intake, we explored the balance of nutrients that could best reflect the characteristics of the group with a high “Healthy food choice” factor score as a potential indicator of nutritional balance improvement. We found that the ratios of major nutrients shown in Table 6 (i.e., protein/fat ratio, dietary fiber/carbohydrate ratio, N-3 fatty acid/fat ratio, and EPA + DHA/fat ratio) are strongly associated with “Healthy food choice” factor scores. In addition, the fish and seafood protein/total animal protein ratio was significantly different, while the plant protein/animal protein ratio was not different, indicating that the increased amounts of protein from fish and seafood played a significant role in “Healthy food choice.” Our findings suggest that visceral fat accumulation can be prevented by adjusting the nutritional balance through dietary instruction to increase these ratios, while maintaining a constant calorie intake from meals.

We then estimated the target ratios of dietary balance (Table 6) within the scope of the 2010 edition of Dietary Reference Intake (DRI) for Japanese [18] which was the latest DRIs of the time when the survey was conducted, specifically from the viewpoint of preventing visceral fat accumulation in Japanese people. Taking into consideration the lower limit of calorie requirement by sex and age for men and women at least 20 years of age, we set 2000 kcal for calorie intake and a target protein/fat ratio of 1.0 (protein, 20%; fat, 20%) to achieve the highest protein/fat ratio within recommended ranges (i.e., protein, 10–20 cal%; fat, 20–30 cal%) [18]. In this scenario, carbohydrate intake is 60 cal% (300 g), and a desirable dietary fiber/carbohydrate ratio of ≥0.063 (dietary fiber, 19 g; carbohydrates, 300 g) can be set to satisfy the target intake of dietary fiber (≥19 g) for all sexes and age groups [18]. Moreover, with a fat intake of 44.4 g, the target N-3 fatty acid/fat ratio is roughly 0.054 (N-3 fatty acids, 2.4 g; fat, 44.4 g) to satisfy the target intake of N-3 fatty acids (≥2.4 g) for both sexes and all age groups [18]. In particular, if we limit N-3 fatty acids to EPA + DHA (target amount ≥1.0 g), a target EPA + DHA/fat ratio of roughly 0.023 (EPA + DHA, 1.0 g; fat, 44.4 g) can be set. Increasing these ratios is expected to contribute positively to the prevention of visceral fat accumulation. Actually, the group with a high “Healthy food choice” factor score (Q4) was higher in these ratios, but less than targeted ratios. Further decrease of fat intake is expected to be effective for improvement of protein/fat ratio and N-3 fatty acid/fat ratio. Increase of dietary fiber intake rather than decrease of carbohydrate intake might be effective for desirable dietary fiber/carbohydrate ratio. The intake of “seasoning and spices” and “salt” tended to increase with this food selection pattern. As Japanese meals are characterized by the custom of using high-salt seasoning such as soy sauce and miso (fermented soybean paste) when cooking “beans,” “vegetables,” and “fish and seafood,” it will be necessary to also consider devising cooking methods, in addition to food selection to reduce salt intake to reach the recommended DRI for sodium.

Physical activity intensity represented by the “Sedentary behavior” factor score was strongly associated with visceral fat accumulation. According to intensity, the group with more moderate to vigorous activities had lower visceral fat accumulation, but no association between walking time and visceral fat was observed in the present study. Ogawara et al. conducted a systematic review of clinical studies on visceral fat reduction by aerobic exercise and reported that at least 10 METs hour/week of aerobic exercise (e.g., active walking, light jogging, ergometer, etc.) is necessary for reducing visceral fat [19]. In this context, the results of the present study might suggest that when the goal is to reduce visceral fat by increasing physical activity, increasing the amount of moderate to vigorous exercise, rather than low intensity exercise such as walking, might be necessary.

The group with a high “Calorie restriction” factor score had significantly more visceral fat. This finding likely reflects the fact that “Calorie restriction” is not the cause of visceral fat accumulation, rather, participants aware of their own obesity were trying to limit calories for the sake of weight loss. The decrease in dietary intake was mainly observed in terms of decreased calorie intake from carbohydrates. In recent years, many studies have reported on low carbohydrate diets for improving obesity [6, 20], and in the present questionnaire-based dietary factor analysis, increase in nutrients with respect to “Appetite” was mainly in the form of carbohydrates. Therefore, in obesity measures focusing on calorie restriction, restricting carbohydrates is likely to lead most directly to reducing calories.

Interestingly, “Irregular mealtime” was associated with visceral fat accumulation, and the actual contribution was characterized by a delayed supper time and skipping breakfast. These irregular dietary habits are considered to reflect characteristic trends of modern society, and in recent years, various studies have been conducted on their relationships with obesity from the perspective of chrononutrition [21]. The results of the present study strongly suggest that dietary habit modification focusing on mealtime is also important from the perspective of visceral fat accumulation.

This study has some limitations. Survey 2 was a personal online survey, and the anthropometric data were self-reported without verification, so the reliability of the entered data was not validated. Due to the cross-sectional design, we could not demonstrate causal relationships. Surveys 1 and 2 were independent cohorts, and a direct relationship between visceral fat accumulation and dietary habits at the individual level could not be discussed. Furthermore, our survey population comprised Japanese people living in urban areas, and therefore reflected neither the influence of different residential areas nor racial and international differences in dietary habits. For these reasons, such validation should be planned as a next step to confirm and validate the findings of the current study.

Despite these limitations, the present study was successful in quantitatively correlating visceral fat accumulation with associated dietary habits in a large study population. Based on the findings of the present study, we are currently developing a method for dietary counseling aimed at prevention and improvement of visceral fat accumulation. In the future, we plan to conduct a prospective study to verify the usefulness of this method.

## 5. Conclusions

A newly developed dietary habit questionnaire allowed the assessment of dietary habits involved in the visceral fat accumulation at an individual level. Moreover, we found correlations between relevant dietary factors and the characteristics of nutritional balance. This questionnaire could be used to provide suggestions for dietary modification to achieve visceral fat reduction on an individual basis.

## Figures and Tables

**Table 1:** Anthropometric parameters of participants in Survey 1 and Survey 2.

	Sex	Age	*n*	Age (years)	BMI (kg/m^2^)	Waist (cm)	VFA (cm^2^)	*t*-test^1^
Survey 1	Male	20–29	1317	26.3 ± 2.0	21.9 ± 2.8	78.1 ± 7.6	56 ± 37	
		30–39	1888	34.5 ± 2.9	23.0 ± 3.1	81.5 ± 8.2	76 ± 42	
		40–49	2810	44.9 ± 2.6	23.6 ± 3.0	84.5 ± 8.0	90 ± 42	
		50–59	2046	54.1 ± 2.9	23.7 ± 2.8	85.5 ± 8.0	95 ± 41	
		60+	311	61.9 ± 2.3	23.7 ± 2.4	86.2 ± 6.4	100 ± 37	
	Female	20–29	678	26.0 ± 2.1	20.0 ± 2.5	72.0 ± 6.4	33 ± 19	
		30–39	1029	34.6 ± 2.9	20.5 ± 2.6	74.4 ± 7.0	39 ± 21	
		40–49	1029	43.9 ± 2.7	21.1 ± 3.0	77.0 ± 8.1	47 ± 26	
		50–59	300	53.4 ± 2.7	21.5 ± 3.0	78.8 ± 8.6	54 ± 28	
		60+	30	62.0 ± 1.6	22.7 ± 3.1	82.8 ± 10.6	68 ± 36	

Survey 2	Male	30–39	113	35.2 ± 3.0	23.6 ± 3.2	83.0 ± 9.3		0.028
		40–49	118	44.3 ± 2.9	24.0 ± 2.9	84.2 ± 7.6		n.s.
		50–59	68	53.1 ± 2.7	23.8 ± 3.5	86.2 ± 8.2		n.s.
		60+	75	63.5 ± 3.0	23.4 ± 2.3	87.1 ± 6.6		n.s.
	Female	30–39	38	34.4 ± 3.0	20.6 ± 3.4	70.5 ± 8.1		n.s.
		40–49	42	43.8 ± 2.6	21.5 ± 2.8	72.8 ± 8.6		n.s.
		50–59	72	53.4 ± 2.5	21.4 ± 3.6	73.5 ± 10.4		n.s.
		60+	53	63.0 ± 2.7	22.4 ± 3.5	78.8 ± 11.1		n.s.

Mean ± SD. BMI: body mass index; VFA: visceral fat area. ^1^Differences in BMI in each sex and age group between Survey 1 and Survey 2.

**Table 2:** Factor analysis of 35 dietary habit questions in Survey 1 and Survey 2.

	Appetite	Healthy food choice	Sedentary behavior	Calorie restriction	Irregular mealtime
Eigenvalue	3.341	1.874	1.803	1.661	1.643
% of variance	9.547	5.354	5.151	4.746	4.695
Cumulative % of variance	9.547	14.900	20.051	24.797	29.492
If someone around me is eating, I end up eating with them	0.763	−0.046	0.059	0.039	0.044
Even if I'm not hungry, if it smells good, I eat it	0.746	−0.021	0.045	0.037	0.036
I can't help myself from eating when fruits or snacks are out	0.686	−0.094	0.026	0.043	0.010
I eat anything that people give me because otherwise I feel that it's wasteful	0.551	−0.028	−0.008	0.043	0.073
I overeat when I am depressed	0.533	0.012	0.042	0.212	0.083
I don't have a sweet tooth	0.523	−0.080	0.019	0.084	−0.033
I feel uneasy if I don't buy more than what is needed for food	0.420	0.069	0.084	0.071	0.063
I like hamburgers, donuts, and potato chips	0.329	−0.281	0.044	0.024	0.057
I think that I will be miserable if I don't eat	0.319	0.026	−0.023	−0.064	−0.008
I feel like I'm hungry all day	0.309	0.016	−0.026	0.180	0.044
I have late night snacks	0.261	−0.057	0.028	−0.045	0.261
I often have a late night meal after dinner	0.245	−0.003	0.033	−0.041	0.121
I get nervous easily	0.169	−0.021	0.081	0.018	0.011
I hardly chew my food at all	0.128	−0.126	0.145	0.015	0.087
I think I eat faster than others	0.099	−0.016	−0.025	0.089	0.023
I proactively choose foods with lots of dietary fiber	0.071	0.666	−0.131	0.248	−0.103
I try to hold off on animal fats and instead get plant-based or fish fats	−0.049	0.653	−0.046	0.219	−0.059
I am proactive in eating green and yellow vegetables	0.073	0.636	−0.145	0.171	−0.106
I like fish more than meat	−0.074	0.485	−0.003	0.025	0.007
I am cooperative	0.074	0.085	−0.011	0.005	0.003
I don't exercise much	0.024	−0.038	0.901	−0.065	0.022
I think I am more out of shape than overeating	0.015	−0.016	0.728	−0.024	0.021
I don't like walking or biking	0.032	−0.143	0.393	0.028	0.038
If there is an elevator or escalator I use it	0.124	−0.174	0.313	−0.046	0.088
I am lazy	0.210	−0.119	0.241	−0.016	0.088
I am proactive	0.002	0.085	−0.119	0.041	0.005
I consciously hold back from eating too much to avoid weight gain	−0.036	0.167	−0.093	0.661	−0.081
I eat less because I feel guilty about overeating	0.126	0.065	−0.012	0.639	−0.021
I try to buy lower calorie foods	0.070	0.303	−0.033	0.609	−0.035
I think I tend to gain weight more readily than others	0.266	0.059	0.053	0.410	0.038
I am insightful	−0.035	0.042	−0.005	0.069	−0.025
I often have a delayed dinner because of work	0.091	−0.040	0.040	0.001	0.813
My mealtimes are irregular	0.106	−0.120	0.079	0.018	0.646
I am a night owl who doesn't do well in the morning	0.081	−0.112	0.165	−0.031	0.190
I finish dinner 2 or more hours earlier than bedtime	−0.019	0.045	−0.013	0.089	−0.599

**Table 3:** Comparison of dietary factor score between survey, sex, and age groups.

		Appetite	Healthy food choice	Sedentary behavior	Calorie restriction	Irregular mealtime
Dietary factor score	Survey 1	−0.01 ± 0.92	−0.01 ± 0.86	0.00 ± 0.93	0.00 ± 0.84	0.02 ± 0.88
	Survey 2	0.14 ± 0.75	0.16 ± 0.67	−0.03 ± 0.73	0.03 ± 0.66	−0.37 ± 0.77

*p* value^1^	Survey	0.015	0.876	0.692	0.118	0.296
	Sex	0.000	0.000	0.000	0.229	0.000
	Age	0.000	0.000	0.010	0.008	0.000

Mean ± SD. ^1^Analysis of variance fixed effect: survey, sex, and age group.

**Table 4:** Estimated BMI and visceral fat area by quartiles of each dietary factor score in Survey 1.

		Q1	Q2	Q3	Q4	ANOVA^1^	Q1 vs. Q4
Appetite	Male / Female	2468 / 445	2241 / 595	2058 / 779	1605 / 1247		
	Age	44.1 ± 0.2	42.4 ± 0.2	40.5 ± 0.2	38.1 ± 0.2		
	BMI^2^	21.6 ± 0.1	22.2 ± 0.1	22.9 ± 0.1	23.5 ± 0.1	<0.001	<0.001
	Visceral fat^2^	63 ± 1	68 ± 1	76 ± 1	81 ± 1	<0.001	<0.001

Healthy food choice	Male / Female	2324 / 604	2099 / 749	2015 / 794	1934 / 919		
	Age	37.7 ± 0.2	40.4 ± 0.2	42.6 ± 0.2	44.6 ± 0.2		
	BMI	22.5 ± 0.1	22.7 ± 0.1	22.6 ± 0.1	22.5 ± 0.1	0.016	n.s.
	Visceral fat	72 ± 1	74 ± 1	72 ± 1	69 ± 1	<0.01	<0.01

Sedentary behavior	Male / Female	2374 / 529	2141 / 644	2016 / 826	1841 / 1067		
	Age	41.9 ± 0.2	42.0 ± 0.2	40.8 ± 0.2	40.5 ± 0.2		
	BMI^2^	22.1 ± 0.1	22.7 ± 0.1	22.7 ± 0.1	22.8 ± 0.1	<0.001	<0.001
	Visceral fat^2^	58 ± 1	73 ± 1	76 ± 1	81 ± 1	<0.001	<0.001

Calorie restriction	Male / Female	2255 / 657	2055 / 730	2074 / 782	1988 / 897		
	Age	40.2 ± 0.2	41.8 ± 0.2	42.1 ± 0.2	41.2 ± 0.2		
	BMI^2^	21.5 ± 0.1	22.6 ± 0.1	22.9 ± 0.1	23.2 ± 0.1	<0.001	<0.001
	Visceral fat^2^	62 ± 1	74 ± 1	76 ± 1	76 ± 1	<0.001	<0.001

Irregular mealtime	Male / Female	1782 / 1004	2016 / 799	2200 / 701	2374 / 562		
	Age	43.3 ± 0.2	42.4 ± 0.2	40.7 ± 0.2	38.8 ± 0.2		
	BMI^2^	22.3 ± 0.1	22.4 ± 0.1	22.7 ± 0.1	22.9 ± 0.1	<0.001	<0.001
	Visceral fat^2^	68 ± 1	70 ± 1	74 ± 1	76 ± 1	<0.001	<0.001

Mean ± SE. ^1^Fixed effect: quartiles of each dietary factor score; covariates: sex and age. ^2^Data were log-transformed for ANOVA.

**Table 5:** Dietary assessment across quartiles of “Appetite” score in Survey 2.

“Appetite” score quartile		Q1	Q2	Q3	Q4	ANOVA^1^	Q1 vs. Q4
Male / Female		81 / 11	120 / 48	107 / 60	66 / 86		
Age (years)		48.2 ± 1.1	49.5 ± 0.9	47.6 ± 0.8	47.4 ± 0.9		
Grains	g	364 ± 11	369 ± 8	388 ± 8	386 ± 9	n.s.	n.s.
Potatoes	g	26 ± 4	27 ± 3	20 ± 3	28 ± 3	n.s.	n.s.
Sugar and sweeteners	g	6 ± 1	7 ± 1	7 ± 1	8 ± 1	n.s.	0.040
Beans^2^	g	47 ± 5	49 ± 4	49 ± 4	48 ± 4	n.s.	n.s.
Nuts	g	2 ± 0	1 ± 0	1 ± 0	2 ± 0	n.s.	n.s.
Vegetables	g	236 ± 13	255 ± 10	240 ± 9	246 ± 10	n.s.	n.s.
Fruits^2^	g	56 ± 9	62 ± 7	78 ± 7	76 ± 7	n.s.	n.s.
Mushrooms^2^	g	7 ± 1	7 ± 1	8 ± 1	5 ± 1	0.041	n.s.
Seaweed^2^	g	4 ± 1	6 ± 1	4 ± 1	4 ± 1	n.s.	n.s.
Fish and seafood	g	60 ± 4	54 ± 3	54 ± 3	56 ± 3	n.s.	n.s.
Meat	g	82 ± 5	82 ± 3	79 ± 3	85 ± 4	n.s.	n.s.
Egg	g	30 ± 3	34 ± 2	36 ± 2	38 ± 2	n.s.	0.048
Milk products	g	91 ± 10	102 ± 7	93 ± 7	103 ± 7	n.s.	n.s.
Edible oil^2^	g	15 ± 1	17 ± 1	15 ± 1	15 ± 1	n.s.	n.s.
Snack^2^	g	12 ± 3	13 ± 2	22 ± 2	27 ± 2	n.s.	n.s.
Beverage	g	213 ± 25	209 ± 18	265 ± 18	271 ± 20	0.050	n.s.
Seasoning and spices	g	164 ± 11	182 ± 8	182 ± 8	168 ± 9	n.s.	n.s.
Processed foods	g	14 ± 3	17 ± 2	12 ± 2	12 ± 2	n.s.	n.s.
Energy	kcal	1710 ± 37	1773 ± 27	1783 ± 27	1870 ± 29	<0.01	<0.001
Protein	kcal	263 ± 6	262 ± 5	262 ± 5	273 ± 5	n.s.	n.s.
Fat	kcal	523 ± 17	550 ± 12	526 ± 12	564 ± 13	n.s.	0.041
Carbohydrate	kcal	881 ± 21	914 ± 16	947 ± 16	975 ± 17	<0.01	<0.01
Alcohol	kcal	26 ± 8	32 ± 6	31 ± 6	44 ± 7	n.s.	n.s.

Mean ± SE. ^1^Fixed effect: “Appetite” score quartiles; covariates: sex and age. ^2^Data were log-transformed for ANOVA.

**Table 6:** Dietary assessment across quartiles of “Healthy food choice” score in Survey 2.

“Healthy food choice” score quartile		Q1	Q2	Q3	Q4	ANOVA^1^	Q1 vs. Q4
Male / Female		65 / 12	108 / 48	115 / 80	86 / 65		
Age (years)		43.6 ± 1.1	46.5 ± 0.8	48.5 ± 0.8	51.9 ± 0.9		
Grains	g	385 ± 12	388 ± 9	376 ± 8	367 ± 9	n.s.	n.s.
Potatoes	g	28 ± 4	25 ± 3	26 ± 2	24 ± 3	n.s.	n.s.
Sugar and sweeteners	g	7 ± 1	7 ± 1	7 ± 1	8 ± 1	n.s.	n.s.
Beans^2^	g	33 ± 5	38 ± 4	48 ± 3	68 ± 4	<0.001	<0.001
Nuts^2^	g	1 ± 0	1 ± 0	1 ± 0	2 ± 0	n.s.	n.s.
Vegetables^2^	g	201 ± 14	218 ± 10	251 ± 9	289 ± 10	<0.001	<0.001
Fruits^2^	g	64 ± 10	51 ± 7	70 ± 6	89 ± 7	n.s.	n.s.
Mushrooms	g	6 ± 1	5 ± 1	7 ± 1	7 ± 1	n.s.	n.s.
Seaweed^2^	g	3 ± 1	3 ± 1	5 ± 1	7 ± 1	<0.01	n.s.
Fish and seafood^2^	g	48 ± 5	49 ± 3	58 ± 3	62 ± 3	0.014	0.039
Meat	g	91 ± 5	79 ± 4	84 ± 3	78 ± 4	n.s.	n.s.
Egg	g	37 ± 3	33 ± 2	36 ± 2	36 ± 2	n.s.	n.s.
Milk products	g	101 ± 10	97 ± 7	95 ± 6	101 ± 7	n.s.	n.s.
Edible oil^2^	g	17 ± 1	14 ± 1	16 ± 1	15 ± 1	n.s.	n.s.
Snack	g	19 ± 3	20 ± 2	18 ± 2	19 ± 2	n.s.	n.s.
Beverage	g	244 ± 28	217 ± 19	257 ± 17	247 ± 20	n.s.	n.s.
Seasoning and spices	g	150 ± 12	169 ± 8	188 ± 7	180 ± 8	0.039	0.017
Processed foods^2^	g	17 ± 3	16 ± 2	13 ± 2	11 ± 2	n.s.	n.s.
Energy	kcal	1791 ± 41	1771 ± 28	1791 ± 25	1814 ± 29	n.s.	n.s.
Protein	kcal	259 ± 7	255 ± 5	266 ± 4	277 ± 5	0.013	n.s.
Fat	kcal	564 ± 18	526 ± 13	547 ± 11	542 ± 13	n.s.	n.s.
Saturated	g	16.7 ± 0.7	15.8 ± 0.4	16.4 ± 0.4	16.0 ± 0.5	n.s.	n.s.
Mono-unsaturated	g	23.5 ± 0.9	20.9 ± 0.6	22.5 ± 0.5	21.5 ± 0.6	n.s.	n.s.
Poly-unsaturated	g	12.3 ± 0.5	11.7 ± 0.3	12.7 ± 0.3	12.8 ± 0.3	n.s.	n.s.
N-3 poly-unsaturated	g	2.0 ± 0.1	2.0 ± 0.1	2.2 ± 0.1	2.2 ± 0.1	0.041	0.047
N-6 poly-unsaturated	g	10.3 ± 0.4	9.7 ± 0.3	10.5 ± 0.3	10.5 ± 0.3	n.s.	n.s.
EPA + DHA	mg	433 ± 60	514 ± 41	567 ± 37	642 ± 43	0.030	<0.01
Cholesterol	mg	312 ± 15	287 ± 11	307 ± 9	309 ± 11	n.s.	n.s.
Carbohydrate	kcal	924 ± 24	940 ± 16	926 ± 15	945 ± 17	n.s.	n.s.
Dietary fiber	g	11.8 ± 0.5	11.8 ± 0.3	12.7 ± 0.3	14.1 ± 0.3	<0.001	<0.01
Salt	g	8.9 ± 0.3	9.3 ± 0.2	9.6 ± 0.2	9.9 ± 0.2	0.031	<0.01
Alcohol	kcal	23.9 ± 9.1	28.9 ± 6.3	36.5 ± 5.6	41.1 ± 6.5	n.s.	n.s.
Protein / fat	kcal/kcal	0.479 ± 0.015	0.506 ± 0.010	0.509 ± 0.009	0.535 ± 0.011	0.026	<0.01
Dietary fiber / carbohydrate	g/g	0.052 ± 0.002	0.051 ± 0.001	0.056 ± 0.001	0.060 ± 0.001	<0.001	<0.01
N-3 fatty acid / fat	g/g	0.031 ± 0.002	0.034 ± 0.001	0.036 ± 0.001	0.038 ± 0.001	<0.01	<0.001
EPA + DHA / fat	g/g	0.007 ± 0.001	0.009 ± 0.001	0.010 ± 0.001	0.011 ± 0.001	0.012	<0.01
Plant protein / animal protein^2^	g/g	1.162 ± 0.083	1.090 ± 0.058	0.982 ± 0.051	1.151 ± 0.059	n.s.	n.s.
Fish and Seafood protein / total animal protein	g/g	0.254 ± 0.021	0.282 ± 0.015	0.323 ± 0.013	0.338 ± 0.015	<0.01	<0.01

Mean ± SE. ^1^Fixed effect: “Healthy food choice” score quartiles; covariates: sex and age. ^2^Data were log-transformed for ANOVA.

**Table 7:** Quartiles of other dietary factor scores in Survey 2.

“Sedentary behavior” score quartile		Q1	Q2	Q3	Q4	ANOVA^1^	Q1 vs. Q4
Male / Female		70 / 32	140 / 79	106 / 56	58 / 38		
Age (years)		48.4 ± 1.1	49.2 ± 0.7	47.0 ± 0.8	47.8 ± 1.1		
Vigorous (8.0 MET)^4^	min./week	212 ± 12	74 ± 9	17 ± 10	10 ± 13	<0.001	<0.001
Moderate (4.0 MET)^4^	min./week	128 ± 11	66 ± 8	21 ± 9	21 ± 11	<0.001	<0.001
Walking (3.3 MET)	min./week	253 ± 24	247 ± 16	264 ± 19	228 ± 25	n.s.	n.s.
Total MET-min./week		3045 ± 144	1670 ± 98	1091 ± 114	915 ± 148	<0.001	<0.001
“Calorie restriction” score quartile		Q1	Q2	Q3	Q4	ANOVA^2^	Q1 vs. Q4
Male / Female		57 / 36	131 / 88	113 / 35	73 / 46		
Age		46.7 ± 1.2	48.7 ± 0.7	48.6 ± 0.9	48.0 ± 0.9		
Energy	kcal	1831 ± 37	1811 ± 24	1791 ± 29	1727 ± 32	n.s.	0.042
Protein	kcal	265 ± 6	267 ± 4	267 ± 5	258 ± 5	n.s.	n.s.
Fat	kcal	540 ± 16	547 ± 11	552 ± 13	523 ± 14	n.s.	n.s.
Carbohydrate	kcal	978 ± 21	942 ± 14	918 ± 17	905 ± 18	0.045	0.015
Alcohol	kcal	30 ± 8	36 ± 5	40 ± 6	27 ± 7	n.s.	n.s.
“Irregular mealtime” score quartile		Q1	Q2	Q3	Q4	ANOVA^3^	Q1 vs. Q4
Male / Female		105 / 114	119 / 70	87 / 16	63 / 5		
Age (years)		51.3 ± 0.8	48.7 ± 0.7	44.4 ± 0.8	42.4 ± 1.0		
Breakfast	hh:mm	7:36 ± 0:03	7:33 ± 0:04	7:45 ± 0:05	7:33 ± 0:07	n.s.	n.s.
Lunch	hh:mm	12:34 ± 0:02	12:31 ± 0:02	12:43 ± 0:04	12:41 ± 0:05	n.s.	n.s.
Supper^4^	hh:mm	19:12 ± 0:04	19:29 ± 0:04	20:06 ± 0:06	20:35 ± 0:08	<0.001	<0.001
Skip breakfast^4^	%	4 ± 1	7 ± 1	9 ± 2	22 ± 2	<0.001	<0.001
Skip lunch^4^	%	6 ± 1	4 ± 1	9 ± 2	6 ± 2	n.s.	n.s.
Skip supper^4^	%	2 ± 1	3 ± 1	3 ± 1	4 ± 1	n.s.	n.s.

Mean ± SE. ^1^Fixed effect: “Sedentary behavior” score quartiles; covariates: sex and age. ^2^Fixed effect: “Calorie restriction” score quartiles; covariates: sex and age. ^3^Fixed effect: “Irregular mealtime” score quartiles; covariates: sex and age. ^4^Data were log-transformed for ANOVA.

## Data Availability

The datasets analyzed during the current study are available from the corresponding author on reasonable request.
